# A Multifactorial Case of Retroperitoneal Fibrosis in a Post-radiation Bladder Cancer Patient: Diagnostic and Therapeutic Challenges

**DOI:** 10.7759/cureus.89976

**Published:** 2025-08-13

**Authors:** Mohammed Ashrf Abdulhafid Shebani, Ahmed Ali Ikdees, Sirajaldeen Yousuf Alazeebi, Esraa Nagib, Heba M. K. Alagha

**Affiliations:** 1 Internal Medicine, University of Tripoli, Tripoli, LBY; 2 Internal Medicine, Tripoli Central Hospital, Tripoli, LBY

**Keywords:** autoimmune, bladder cancer, radiation therapy, retroperitoneal fibrosis, ureteric obstruction

## Abstract

Retroperitoneal fibrosis (RPF) is an uncommon fibroinflammatory condition characterized by the development of dense fibrotic tissue in the retroperitoneum, often encasing nearby structures such as the ureters, gastrointestinal tract, and blood vessels. It can lead to progressive obstruction of the urinary and gastrointestinal tracts, with clinical manifestations varying depending on the extent and location of involvement. We present a diagnostically challenging case of secondary RPF, most likely triggered by post-radiation changes and autoimmune mechanisms, in a 44-year-old male with a history of high-grade muscle-invasive urothelial carcinoma of the bladder with squamous differentiation, treated with chemotherapy and pelvic radiotherapy. The patient presented with progressive jaundice, abdominal pain, vomiting, and weight loss. Imaging studies, including computed tomography (CT), magnetic resonance imaging (MRI) of the abdomen and pelvis, and magnetic resonance cholangiopancreatography (MRCP), revealed extensive RPF with bilateral ureteric involvement, common bile duct stricture, pyloric narrowing, and colonic stenosis. Laboratory results showed elevated inflammatory markers and a positive antinuclear antibody (ANA) titer. Endoscopic interventions were unsuccessful due to pyloric obstruction. The patient ultimately required bilateral ureteric stenting, percutaneous biliary drainage, and corticosteroid therapy, which led to clinical improvement. The coexistence of post-radiation fibrosis, positive ANA titer (1:160), and previous malignancy-related changes was considered a contributory factor. This case highlights the importance of multidisciplinary management and early intervention in complex presentations of RPF involving oncologic and autoimmune factors.

## Introduction

Retroperitoneal fibrosis (RPF) is a rare fibroinflammatory disorder characterized by the proliferation of dense connective tissue in the retroperitoneal space, frequently leading to compression or obstruction of adjacent structures such as the ureters, inferior vena cava, aorta, and gastrointestinal tract [1]. Although its overall incidence is estimated at only one per 200,000 to 500,000 people annually, secondary forms, particularly those arising after pelvic or abdominal radiotherapy, are exceedingly uncommon and often under-recognized. When secondary to prior radiation, the clinical course can be more insidious, with overlapping features of post-treatment changes, neoplastic recurrence, and chronic inflammation that complicate timely diagnosis.

RPF most commonly affects middle-aged individuals between 40 and 60 years, with a male predominance (male-to-female ratio approximately 2:1 to 3:1). Approximately 70% of cases are idiopathic, often referred to as Ormond’s disease, and are thought to involve an autoimmune-mediated inflammatory response. By contrast, secondary RPF has diverse etiologies, including malignancies, abdominal or pelvic surgery, infections (e.g., tuberculosis, actinomycosis), medications such as methysergide, ergot derivatives, beta-blockers, and certain biologics, as well as prior radiotherapy [2]. Radiation-induced fibrosis is believed to result from chronic microvascular damage, persistent low-grade inflammation, and excessive fibroblast activation, creating a dense collagen matrix that can entrap retroperitoneal structures.

The diagnosis of RPF, especially its secondary forms, can be challenging because early symptoms are nonspecific, such as dull abdominal or back pain, weight loss, or signs of obstructive uropathy. These manifestations are easily misattributed to more common conditions, particularly in patients with complex oncologic or autoimmune backgrounds. Radiological imaging is central to diagnosis: contrast-enhanced computed tomography (CT) and magnetic resonance imaging (MRI) allow assessment of the extent, density, and enhancement patterns of the mass, while magnetic resonance cholangiopancreatography (MRCP) may be useful when biliary involvement is suspected [3].

Here, we report a diagnostically challenging case of secondary RPF in a patient with prior pelvic radiotherapy, a history of malignancy, and positive autoimmune markers. This multifactorial presentation exemplifies the complexity of differentiating between post-radiation fibrosis, recurrent malignancy, and immune-mediated disease and underscores the importance of a multidisciplinary diagnostic approach.

## Case presentation

A 44-year-old Libyan man with a history of high-grade muscle-invasive urothelial carcinoma of the urinary bladder with squamous differentiation - treated with gemcitabine and cisplatin chemotherapy followed by pelvic radiotherapy (total dose 72 Gy in 40 fractions) in 2023 - presented with persistent vomiting, diffuse abdominal and back pain, progressive jaundice, and unintentional weight loss of 6 kg over three months (body mass index (BMI) 16.4 kg/m²). His symptoms had markedly worsened over the preceding two weeks. The last positron emission tomography (PET) scan, performed overseas eight months prior, demonstrated marked regression of the primary tumor with complete metabolic clearance. 

On examination, the patient appeared acutely ill, deeply jaundiced, cachectic, and pale, without peripheral stigmata of chronic liver disease. Vital signs were as follows: BP 100/65 mmHg, HR 80 bpm, and temperature 37 °C. The abdomen was distended and diffusely tender with sluggish bowel sounds, but there were no signs of peritonitis or portal hypertension.

Laboratory and imaging findings

Laboratory investigations revealed mild anemia, normal leukocyte and platelet counts, a cholestatic liver enzyme pattern, and significantly elevated inflammatory markers. Autoimmune screening demonstrated a positive antinuclear antibody (ANA) titer of 1:160. Serum IgG4 concentration was within normal limits. Pancreatic enzymes (amylase and lipase), thyroid function tests (TSH, free T4), and thyroid autoantibodies (anti-thyroid peroxidase, anti-thyroglobulin) were normal. A detailed summary of laboratory values is presented in Table 1.

**Table 1:** Summary of laboratory investigations on admission Values are shown alongside reference ranges for comparison. Notable findings included a cholestatic liver enzyme pattern, elevated inflammatory markers, and a positive (ANA) titer.

Parameter	Result	Reference range
Hemoglobin (Hb)	12 g/dL	13.0–17.0 g/dL (males)
White blood cell count (WBC)	6.28 × 10⁹/L	4.0–11.0 × 10⁹/L
Platelet count (PLT)	384 × 10⁹/L	150–400 × 10⁹/L
Blood urea	56 mg/dL	10–50 mg/dL
Serum creatinine	0.7 mg/dL	0.6–1.3 mg/dL
Aspartate aminotransferase (AST)	72 IU/L	10–40 IU/L
Alanine aminotransferase (ALT)	63 IU/L	10–45 IU/L
Alkaline phosphatase (ALP)	525 IU/L	40–130 IU/L
Total bilirubin	35 µmol/L	5–21 µmol/L
Direct (conjugated) bilirubin	28 µmol/L	<5 µmol/L
Serum albumin	3 g/dL	3.5–5.0 g/dL
Erythrocyte sedimentation rate (ESR)	90 mm/hr	<20 mm/hr
C-reactive protein (CRP)	17 mg/L	<5 mg/L
Antinuclear antibody (ANA)	Positive (1:160)	Negative
Immunoglobulin G4 (IgG4)	0.13 g/L	0.03–2.01 g/L

These findings, in the context of the patient’s clinical presentation and imaging results, raised the suspicion of an inflammatory or fibrotic retroperitoneal process with possible autoimmune involvement.

**Figure 1:**
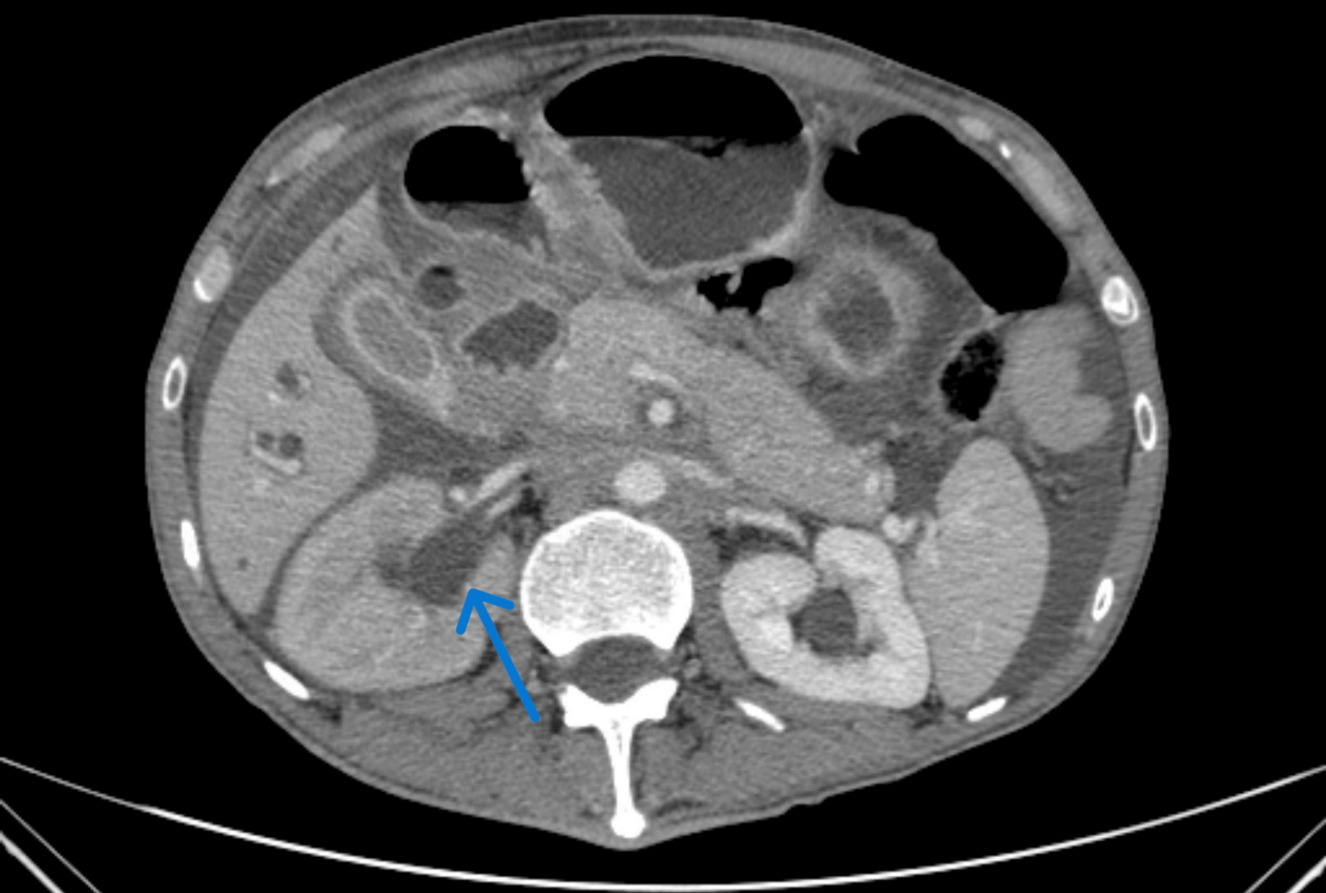
A contrast-enhanced CT of the abdomen and pelvis in September 2024 demonstrated a right hydronephrosis, thickening of the transverse colon, and gallbladder thickening with pericholecystic inflammation.

As shown in Figure 1, a contrast-enhanced CT scan of the abdomen and pelvis shows a right ureteric encasement and moderate right hydronephrosis.

**Figure 2:**
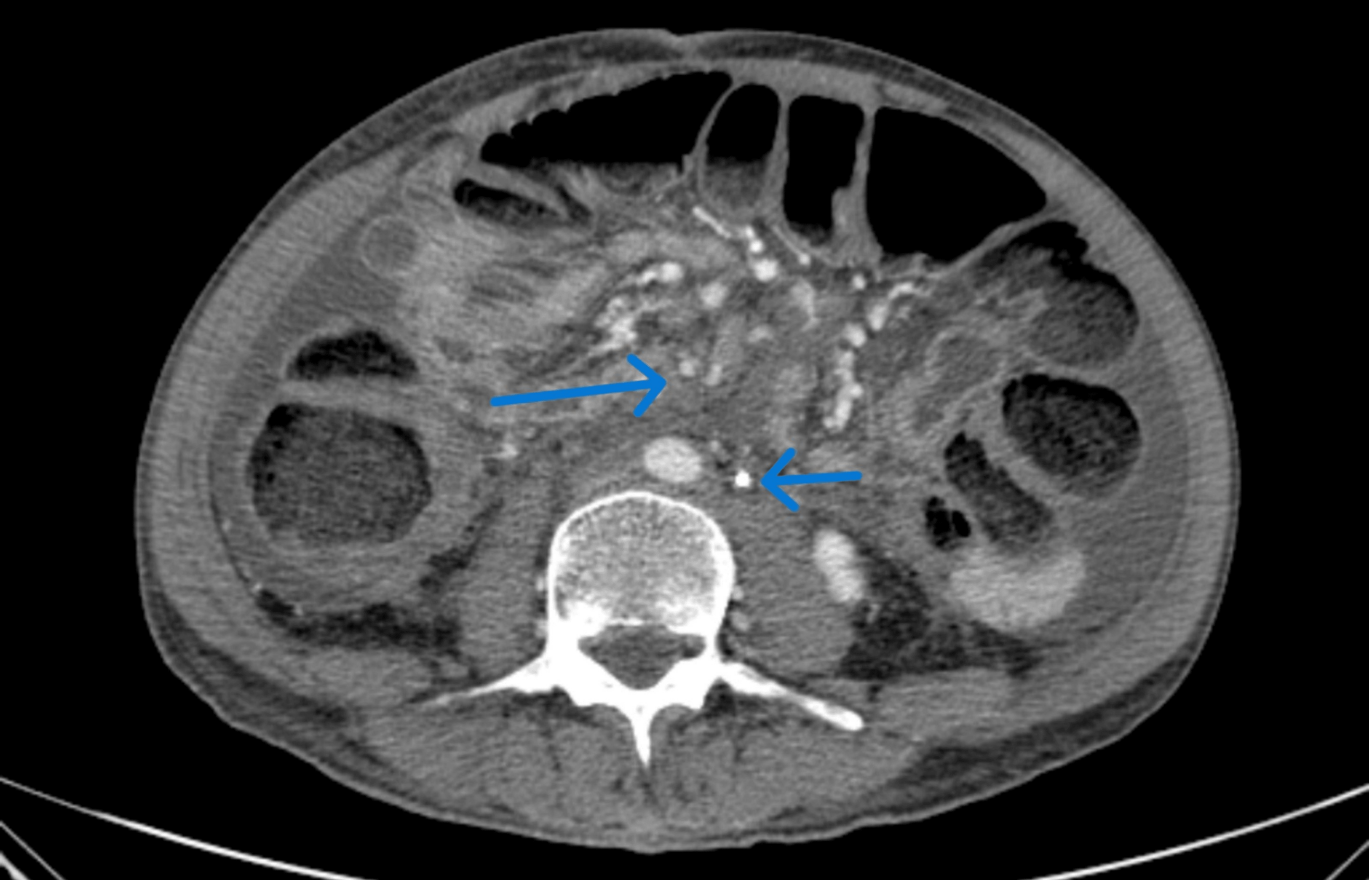
Axial contrast-enhanced CT scan showing retroperitoneal soft tissue encasing the abdominal aorta and bilateral ureters, consistent with retroperitoneal fibrosis.

An MRCP, performed 10 days later in October 2024, confirmed the narrowing of the common bile duct with upstream dilation, and as shown in Figure 2, further CT imaging revealed features consistent with RPF.

Endoscopic findings

Attempts at upper gastrointestinal endoscopy and colonoscopy were unsuccessful due to two distinct obstructive lesions - a severe pyloric stenosis and a separate tight colonic stricture located approximately 30 cm from the anal verge. Such gastrointestinal involvement, particularly affecting both stomach and colon, is rare in RPF and warrants further discussion in light of existing literature.

Additional evaluations

Cystoscopy findings were normal, with no evidence of tumor recurrence. Peritoneal fluid analysis revealed an exudative effusion without malignant cells on cytology.

Intervention and management

Due to the failure of endoscopic biliary drainage secondary to pyloric obstruction, the patient underwent percutaneous transhepatic biliary drainage, followed by bilateral ureteric double J stent placement. The intestinal obstruction was managed conservatively with nasogastric decompression and supportive care. Symptomatic improvement, including partial resolution of obstructive symptoms, was noted following initiation of high-dose corticosteroid therapy.

## Discussion

This case illustrates the diagnostic complexity of RPF in a patient with multiple overlapping risk factors and unusual multi-organ involvement. From a clinical standpoint, the patient’s presentation with progressive vomiting, jaundice, back pain, weight loss, and cachexia immediately raised concern for a malignant, autoimmune, or fibro-inflammatory process affecting both the gastrointestinal and hepatobiliary systems. The coexistence of obstructive jaundice and severe gastrointestinal obstruction is atypical for RPF, prompting a broad differential diagnosis that included recurrent bladder carcinoma, radiation-induced fibrosis, IgG4-related disease, and other systemic autoimmune disorders.

Secondary causes of RPF are well-documented and include prior malignancies, radiotherapy, surgery, infections, and certain medications such as methysergide, ergot derivatives, beta-blockers, hydralazine, and selected biologic agents [4,5]. In our patient, the most plausible triggers were pelvic radiotherapy and prior urothelial carcinoma, with additional possible contribution from an autoimmune mechanism suggested by the positive ANA titer and markedly elevated inflammatory markers.

Several autoimmune diseases, such as systemic lupus erythematosus, rheumatoid arthritis, autoimmune thyroiditis (Hashimoto’s), and vasculitis, have been reported in association with RPF [2]. These were excluded clinically and through targeted laboratory testing, including normal thyroid function, normal thyroid autoantibodies, and no clinical features suggestive of systemic rheumatologic disease.

Imaging findings demonstrated an extensive fibro-inflammatory process involving bilateral ureters, the distal common bile duct, stomach, and colon (Table 2). Such gastrointestinal tract involvement, especially affecting both pyloric and colonic segments, is rare and likely represents an unusually aggressive form of secondary RPF [3]. Multifocal distribution, in this context, favored a systemic inflammatory process over isolated localized disease.

**Table 2:** Organ involvement in retroperitoneal fibrosis and relevance to the current case CT/MRI: computed tomography/magnetic resonance imaging, MRCP: magnetic resonance cholangiopancreatography

Organ/system involved	Typical clinical manifestations	Imaging findings
Ureters	Flank pain, hydronephrosis, obstructive uropathy	Medial deviation and encasement on CT/MRI, hydronephrosis
Common bile duct	Jaundice, cholestatic liver enzyme elevation	Ductal narrowing/stricture on MRCP or CT
Stomach	Early satiety, nausea, vomiting	Extrinsic compression or displacement on imaging
Colon	Constipation, bowel obstruction, abdominal pain	Segmental narrowing and obstruction on CT

Although a PET scan is recommended in selected cases to distinguish active inflammatory disease from malignancy and to identify potential biopsy targets, the last PET scan, performed overseas eight months prior, showed complete metabolic clearance. A biopsy was considered but was declined by the patient. This limitation necessitated reliance on clinical, laboratory, and radiological features for diagnosis.

Non-invasive radiologic surrogates, including contrast-enhanced CT features of homogeneous soft-tissue encasement without invasive margins and stability over short-interval imaging, supported a benign fibro-inflammatory process.

Although the patient’s serum IgG4 level was 0.13 g/L, it remained well below the threshold typically considered suggestive of IgG4-related disease (IgG4-RD), which is >1.35 g/L [6]. IgG4-RD is a distinct clinicopathologic entity that accounts for a subset of RPF cases and is characterized by systemic fibro-inflammatory involvement, often affecting the pancreas, kidneys, salivary glands, and retroperitoneum. Classic diagnostic features include significantly elevated serum IgG4, storiform fibrosis, obliterative phlebitis, and dense IgG4-positive plasma cell infiltration on histology. While not entirely excludable in the absence of a biopsy, the modest IgG4 elevation in this patient, combined with the presence of a positive ANA, markedly elevated inflammatory markers, and a history of pelvic malignancy and radiotherapy, suggests a more likely autoimmune or post-radiation etiology [7].

Management and outcome

High-dose corticosteroid therapy remains the cornerstone of treatment for idiopathic and many secondary forms of RPF [8]. Our patient was initiated on intravenous methylprednisolone 60 mg daily (due to inability to tolerate oral intake), transitioned to oral prednisone once a soft diet was possible, and tapered gradually over several months to a maintenance dose of 5 mg. Improvement in abdominal pain, resolution of jaundice, and partial restoration of oral intake were noted within weeks of initiation. No steroid-sparing agents were used during this admission. We acknowledge that corticosteroid response is not specific to RPF and may be seen in certain malignancies and other inflammatory conditions; however, in the absence of histological evidence of cancer and in light of sustained clinical improvement, an inflammatory etiology is most likely.

This case underscores the importance of early multidisciplinary intervention, including radiology, gastroenterology, urology, and oncology, to preserve organ function. The combination of ureteral stenting, biliary decompression, nutritional support, and immunosuppression was critical in achieving clinical stabilization. Given the oncologic history, ongoing close surveillance remains essential.

## Conclusions

This case highlights the complex interplay between malignancy-related complications, autoimmune features, and retroperitoneal fibrosis, underscoring the diagnostic and therapeutic challenges clinicians may face in similar scenarios. The patient’s presentation required timely imaging and collaborative input from urology, gastroenterology, and radiology to guide effective intervention and prevent permanent organ damage. In the absence of tissue confirmation, a comprehensive clinical, serological, and radiological assessment was pivotal in formulating a working diagnosis and initiating empiric treatment.

Importantly, this report reinforces the need for increased clinical awareness of secondary RPF in oncology patients, where overlapping features with tumor recurrence can delay diagnosis. A multidisciplinary approach, combining expertise from various specialties, was essential for optimizing outcomes, and empiric immunosuppression played a key role when histologic confirmation was not immediately feasible. Finally, given the patient’s oncologic background and the potential for recurrence or progression, long-term follow-up remains critical to ensure early detection of complications and sustained disease control.
